# Sternal reconstruction with bone cement block using the rigid plate fixation technique

**DOI:** 10.1111/1759-7714.13145

**Published:** 2019-07-09

**Authors:** Jae Jun Jung, Seung Soo Kim, Dong Hoon Kang, Seong Ho Moon, Jun Ho Yang, Joung Hun Byun, Jong Woo Kim, Sung Hwan Kim

**Affiliations:** ^1^ Department of Thoracic and Cardiovascular Surgery, College of Medicine and Institute of Health Sciences, Jinju Gyeongsang National University Changwon Hospital Changwon South Korea; ^2^ Department of Neurosurgery, College of Medicine and Institute of Health Sciences, Jinju Gyeongsang National University Changwon Hospital Changwon South Korea

**Keywords:** Bone cement block, chondrosarcoma, reconstruction

## Abstract

Chest computed tomography demonstrated a suspected primary tumor in the upper sternal body of a 48‐year‐old woman who presented with sternal pain. After being diagnosed with chondrosarcoma, she underwent sternal resection. Subsequent chest wall reconstruction was performed after careful planning using a bone cement block made of polypropylene mesh and polymethylmethacrylate. The block was fixed to the manubrium and ribs using the SternaLock System. She was discharged following an uneventful postoperative recovery, and is currently undergoing follow‐up.

## Introduction

Chondrosarcoma is the most common primary malignant tumor of the sternum.^1^ Because of resistance to chemotherapy and radiation therapy, complete resection is the optimal treatment strategy for these tumors.^2^, ^3^ Material selection and design are important factors in sternal reconstruction. However, appropriate materials are not readily available in all hospitals. Here, we report a case of sternal reconstruction using easily obtainable material.

## Case report

**Figure 1:**
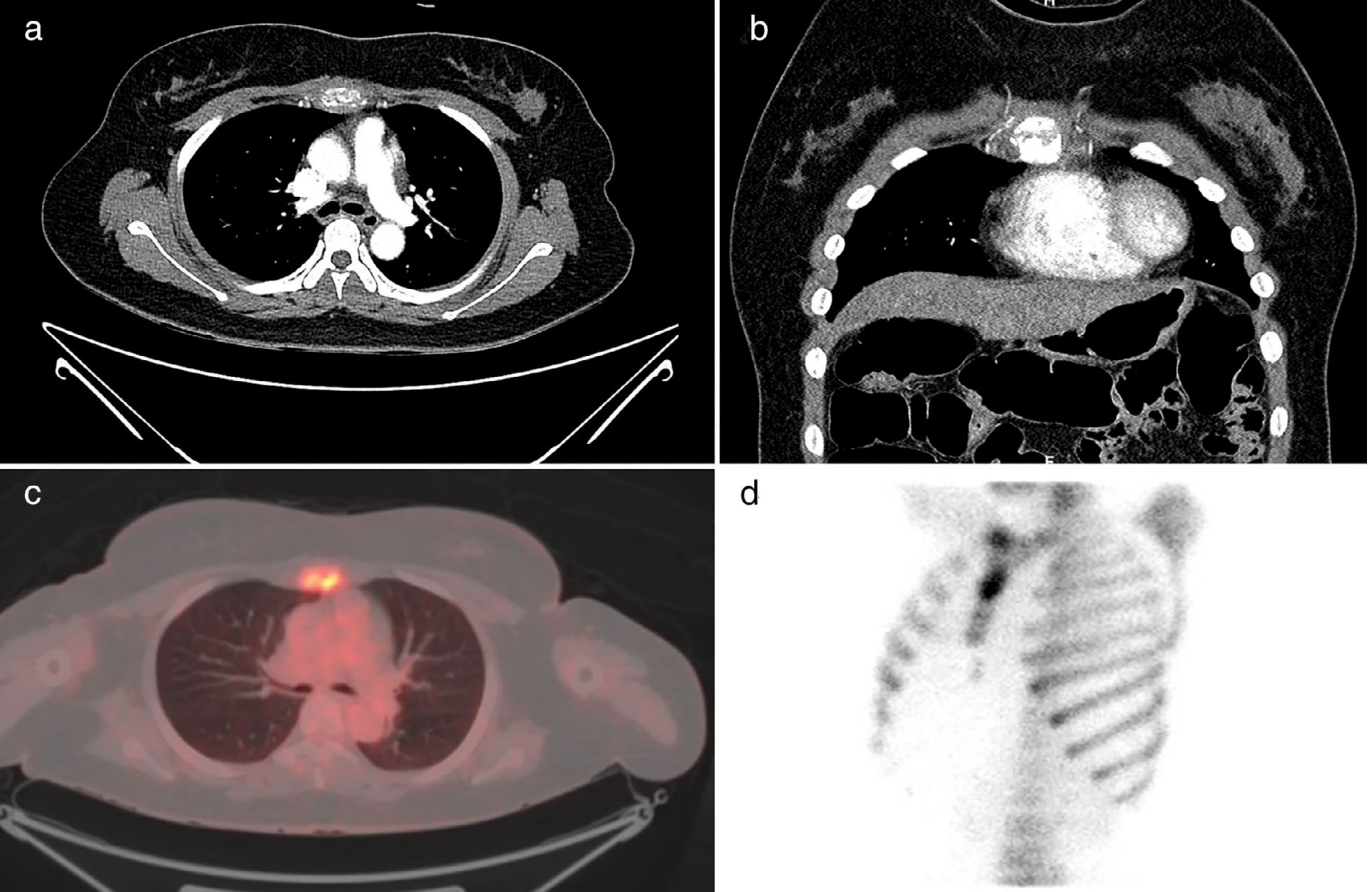
(**a**) Chest CT scan (axial view) showed a mass and a pathological fracture in the upper sternal body. (**b**) Chest CT scan (coronal view) showed a mass and a pathological fracture in the upper sternal body. (**c**) PET‐CT scan showed a heterogeneous hypermetabolic lytic bone lesion in the upper sternal body. (**d**) Bone scan showed increased focal uptake in the upper sternal body. CT, computed tomography; PET, positron emission tomography.

**Figure 2:**
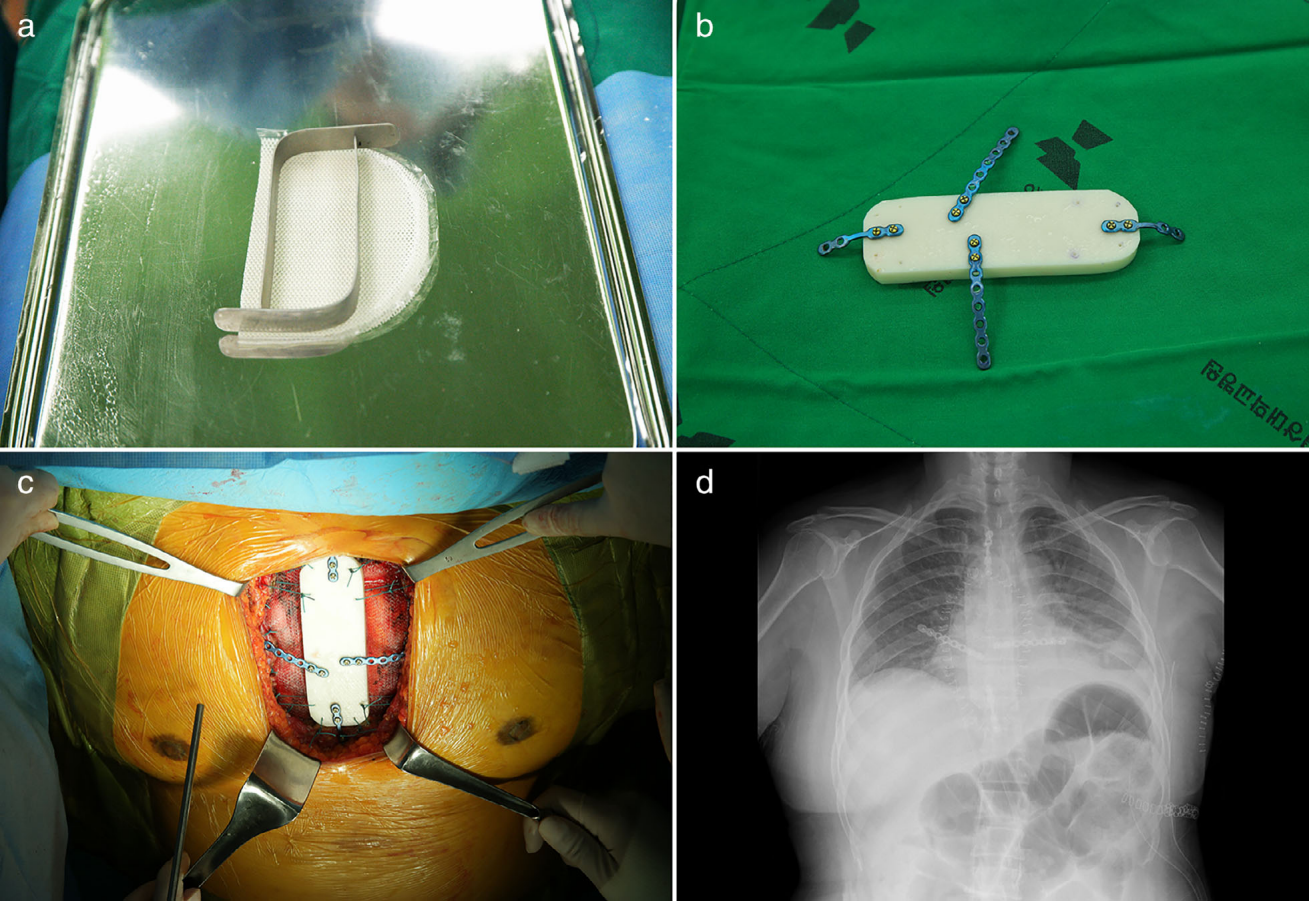
(**a**) The mesh was initially placed on the Mayo stand, and a malleable spatula used to mold the bone cement block. (**b**) After preparing the bone cement, a titanium plate was fixed to the bone cement block, initially to fit the rib structure, and thereafter to fit the remaining sternal structure. (**c**) The neosternum was placed after removal of the sternum. It was fixed to the ribs and the remaining sternum. Ribs 2 and 4 were fixed using 1–0 Ethibond suture. (**d**) Chest radiograph obtained three days postoperatively showed the neosternum constructed using a bone cement block.

A 48‐year‐old woman presented with a three‐week history of sternal pain. Chest computed tomography (CT) revealed a sternal mass and a pathological fracture (Fig 1a,b). Chest magnetic resonance imaging was not feasible as the patient had a first‐grade intellectual disability. Positron emission tomography‐CT revealed hypermetabolic activity with a maximum standardized uptake value of 7.2 in the upper sternal body, without other abnormalities (Fig 1c). Bone scintigraphy revealed increased focal uptake in the upper sternal body (Fig 1d), highly suspicious of a primary sternal tumor. Treatment necessitated sternal resection. A 3 cm vertical incision was performed at the tumor site to expose the sternal cortical bone. Ultrasonography‐guided bone biopsy revealed a diagnosis of bone tumor on frozen section. A wide vertical elliptical incision including the biopsy site was performed over the sternum, extending from the sternal notch to the xiphoid process. The skin and muscle layers were dissected to expose the costochondral junction. Using an oscillating sternal saw, we transected 50% of the manubrium and the lower sternal body. The costochondral junction was transected using electrocautery, and the specimen was removed. After ensuring hemostasis, the pericardium was covered with polypropylene mesh (Marlex, Bard Cardiosurgery, Billerica, MA, USA). Neo‐sternal reconstruction was performed using a polymethylmethacrylate cement block (Antibiotic Simplex P, Howmedica, Limerick, Ireland) and polypropylene mesh (Marlex, Bard Cardiosurgery). The neosternum (cement block) and the remaining bony structures were fixed using the rigid plate‐screw (SternaLock Blu, Zimmer Biomet, Jacksonville, FL, USA) fixation technique. The neosternum and bilateral third ribs were fixed using a rigid plate fixation technique, and bilateral second and fourth ribs were fixed using 1–0 Ethibond sutures (Ethicon, Somerville, NJ, USA) for easy mobility (Fig 2a–d). The skin and muscle layers were reconstructed using a latissimus dorsi myocutaneous flap. The patient was discharged after an uneventful recovery. Chest stability was well‐maintained over a 10‐month follow‐up period. The Human Research Ethics Committee of the Gyeongsang National University Hospital waived the need for approval of this single‐case study. The patient provided written informed consent for the publication of clinical details and images.

## Discussion

Sternal chondrosarcoma is resistant to chemo‐ and radiotherapy. Radical resection to achieve disease‐free margins is the treatment of choice; this is usually performed to the extent that makes sternal reconstruction feasible. Notably, inadequate resection is associated with high recurrence rates. To ensure chest wall stability, total sternectomy should be avoided if the tumor does not involve the entire sternum; subtotal resection is usually recommended.^2^, ^3^

Chest wall reconstruction aims to maintain chest wall stability and rigidity, obliterate dead spaces, preserve pulmonary mechanics (avoiding flail chest), protect intrathoracic organs, provide soft tissue coverage, minimize deformity, and optimize cosmetic outcomes.^2^, ^4^ Sternal reconstruction should be tailored to the extent of soft tissue and skeletal defects. Numerous potentially available combinations of myocutaneous flaps and prostheses are available. However, the optimal approach is usually customized to the individual case.^5^

Previous studies on sternal reconstruction have used various materials,^6^, ^7^, ^8^, ^9^, ^10^ including ‘sandwiched’ polypropylene mesh reinforcement,^6^ individual‐specific stainless steel plates,^7^ titanium mesh,^8^ allogeneic sternal grafts,^9^ and three‐dimensional (3D) printed titanium sternal and rib cages.^10^ Ideally, reconstruction materials should be easily available, easy to handle, durable, resistant to infection, and affordable.^7^

The chest wall muscles and omentum may be used for soft tissue reconstruction. Myocutaneous flaps confer several benefits in terms of safety and long‐term reliability. They may be used alone or in combination, depending on the extent of chest wall defect; the latissimus dorsi, pectoralis major, and rectus abdominis muscles are the most commonly used.^2^ The morbidity and mortality rates of chest wall reconstruction are 24%–66% and 2%–7%, respectively. Pulmonary complications and wound problems are most common.^11^

Polypropylene mesh (Marlex, Bard Cardiosurgery, Billerica, MA), polymethylmethacrylate (Antibiotic SimplexP, Howmedica, Limerick, Ireland), and the rigid plate fixation system (SternaLock Blu, Zimmer Biomet, Jacksonville, FL) were the only materials available at our hospital. The polypropylene mesh covered the pericardium, and the cement block was constructed using polymethylmethacrylate and the mesh. It offers the advantage of moldability based on individual body shapes. The rigid plate fixation system was used to fix the cement block and the remaining sternal and rib structures. Postoperative chest wall stability was maintained, without any complications.

In summary, although a state‐of‐the‐art 3D printed titanium stainless steel prosthesis is useful in such cases, high costs and nonuniform availability hinder its use. Bone cement blocks with rigid plate fixation systems are cost‐effective alternatives for sternal reconstruction following sternal resection.

## Disclosure

The authors report no conflicts of interest.
